# Economic analysis of royalactin production under uncertainty: Evaluating the effect of parameter optimization

**DOI:** 10.1002/btpr.2073

**Published:** 2015-03-15

**Authors:** Mario A. Torres‐Acosta, Jose M. Aguilar‐Yañez, Marco Rito‐Palomares, Nigel J. Titchener‐Hooker

**Affiliations:** ^1^ Centro de Biotecnología‐FEMSA, Tecnológico de Monterrey, Campus MonterreyAve. Eugenio Garza Sada 2501 SurMonterreyNL64849; ^2^ Dept. of Biochemical Engineering, The Advanced Centre for Biochemical EngineeringUniversity College LondonTorrington PlaceLondonWC1E 7JEUK

**Keywords:** royalactin, aqueous two‐phase system (ATPS), economic analysis under uncertainty, Monte Carlo simulation, parameter optimization

## Abstract

Royalactin is a protein with several different potential uses in humans. Research, in insects and in mammalian cells, has shown that it can accelerate cell division and prevent apoptosis. The method of action is through the use of the epidermal growth factor receptor, which is present in humans. Potential use in humans could be to lower cholesterolemic levels in blood, and to elicit similar effects to those seen in bees, e.g., increased lifespan. Mass production of Royalactin has not been accomplished, though a recent article presented a Pichia pastoris fermentation and recovery by aqueous two‐phase systems at laboratory scale as a possible basis for production. Economic modelling is a useful tool with which compare possible outcomes for the production of such a molecule and in particular, to locate areas where additional research is needed and optimization may be required. This study uses the BioSolve software to perform an economic analysis on the scale‐up of the putative process for Royalactin. The key parameters affecting the cost of production were located via a sensitivity analysis and then evaluated by Monte Carlo analysis. Results show that if titer is not optimized the strategy to maintain a low cost of goods is process oriented. After optimization of this parameter the strategy changes to a product‐oriented and the target output becomes the critical parameter determining the cost of goods. This study serves to provide a framework for the evaluation of strategies for future production of Royalactin, by analyzing the factors that influence its cost of manufacture. © 2015 American Institute of Chemical Engineers *Biotechnol. Prog*., 31:744–749, 2015

## Introduction

Royal Jelly (RJ) is the source of nutrition needed to develop bee larvae into future Queen bees. It has a composition of water (60–70%), proteins (12–15%), sugars (10–16%), lipids (3–6%), vitamins, and amino acids.^1^ It is responsible for the epigenetic changes seen during the development of Queen bees: functional reproductive organs, weight increase, and longer lifespan.^2^ Human consumption of RJ has been tested with positive results including reductions in cholesterol and low‐density lipoproteins.^3^, ^4^

Many of the components of RJ have been isolated, characterized, and tested to determine their individual properties. For example, *trans*‐10‐hydroxy‐2‐decenoic acid has been found to possess antibacterial properties,^5^ and Royalisin peptide is active against Gram‐positive bacteria.^6^ From the proteins that compose RJ, around 85% belong to the major RJ proteins (MRJP) 1–5.^7^ Of these proteins, MRJP3 has immunoregulatory effects.^8^ MRJP1, also known as Royalactin, stimulates growth of rat hepatocytes and prevents apoptosis, which suggest action similar to a growth factor.^9^ MRJP1 is also related to the learning ability of bees.^10^ More recently, Royalactin was found to be the only component responsible for the epigenetic changes that larvae undergo to grow as Queen bees, also generating these changes in *Drosophila melanogaster*. Research found that this protein works through binding to the epidermal growth factor receptor (EGFr).^2^ This protein is potentially of great interest for human consumption. Although there is no information on its stability once ingested, there is evidence of decreased cholesterol levels after rat consumption,^11^ thus suggesting Royalactin can withstand gastric conditions. It also helps to explain the anticholesterolemic effects of RJ.^3^, ^4^ Peptides derived from MRJP1 after exposure with trypsin where also tested with positive results in human cell lines.^11^

MRJP1, or Royalactin, is a 57 kDa glycoprotein. It represents approximately 3–4% of total protein in RJ and 30% of MRJPs.^1^ Natural production starts with the collection of RJ. To obtain Royalactin further purification is needed. A well‐trained beekeeper can obtain approximately 500 g of RJ per season (5–6 months),^12^ corresponding to an annual production of almost 35 g of Royalactin. An alternative is the use recombinant organisms. Recombinant production of Royalactin has two main advantages. The first one is the potentially limitless production output and the second one is that MRJP1 can be produced in a pathogen free environment (according to GMP outlines). This is currently critical as the bee population is suffering from colony collapse disorder which has different causes, but includes viral infection from several virus strains.^13^ Although production in recombinant systems is problematic. MRJP1 from European *Apis mellifera* has been produced in *E. coli* with low expression and the formation of inclusion bodies.^14^ In tobacco leaves it has a low titer.^15^ From Eastern *Apis cerana*, MRJP1 has been produced in *E. coli*.^16^ Because of its properties, there is however a great deal of interest in how to achieve mass production of MRJP1. Having a constant supply will allow greater use of the protein in performing assays for food and pharmaceutical applications. Recently production in *Pichia pastoris* with recovery using aqueous two‐phase systems (ATPS) has been reported^17^ as the first attempt to create a bioprocess for the production and recovery of Royalactin.

When designing a bioprocess, it is important to consider possible scenarios that can occur during development. Bioprocess modelling provides a powerful tool to generate a virtual bioprocess, where input parameters, obtained by research, may be used to obtain an estimate of the cost of generating product. Further, use of model‐based techniques can reduce the number of experiments and determine where attention should be focused resulting in reduction of costs, time, and improving decision making.^18^, ^19^ An additional advantage of using modeling is the incorporation of the uncertainties inherent in any bioprocess: changes in production titer, downstream processing yield (DSP), material costs, desired production levels, personnel salary, etc.^19^ This allows the bioprocess engineer to quantify how the production cost varies, and to include the probabilistic nature of these variations when making estimates. This area has gained attention recently, including comparison of the cost of using stainless steel or single‐use equipment,^20^ analysis of the impact that different pooling strategies have in perfusion cultures^21^ and an evaluation of the potential of batch and continuous cell culture technologies.^22^ All these publications identify the best use of technologies and techniques to decrease the cost of production per gram of desired product (cost of goods per gram, CoG/g) without the need to perform extensive experiments, hence saving time by focusing research effort.

Different types of software have been created for bioprocess simulation,^23^, ^24^ including BioSolve (Biopharm Services, Chesham, Buckinghamshire, U.K.), SimBioPharma, Aspen, etc. This software is an Excel‐based modelling tool that takes into account indirect and direct operating costs. This software allows rapid model construction and has the advantage that the costs of equipment and supplies are collected directly from the supplier, which saves time and means that the user can rely on the veracity of the data.

This paper focuses on performing an economic analysis of a bioprocess for the recovery of Royalactin based on the reported production by *Pichia pastoris* and use of ATPS.^17^ This study will focus only on the manufacture of Royalactin. Business related factors were not considered (i.e., product cost, target market, storage, cooling, transportation, etc.). It will study the effect that optimizing a single parameter can have on the CoG/g. For this, a base scale‐up scenario will be designed, from which process parameters will be ranked according to their impact on the CoG/g. A series of Monte Carlo simulations will then be performed in order to incorporate uncertainties inherent in the bioprocess design and hence obtain data on how the CoG/g is distributed. Finally, the simulation approach will be used to develop a proposal on how to achieve a low CoG/g in order to maximize profit.

## Model Set‐Up and Deterministic Analysis

**Figure 1:**
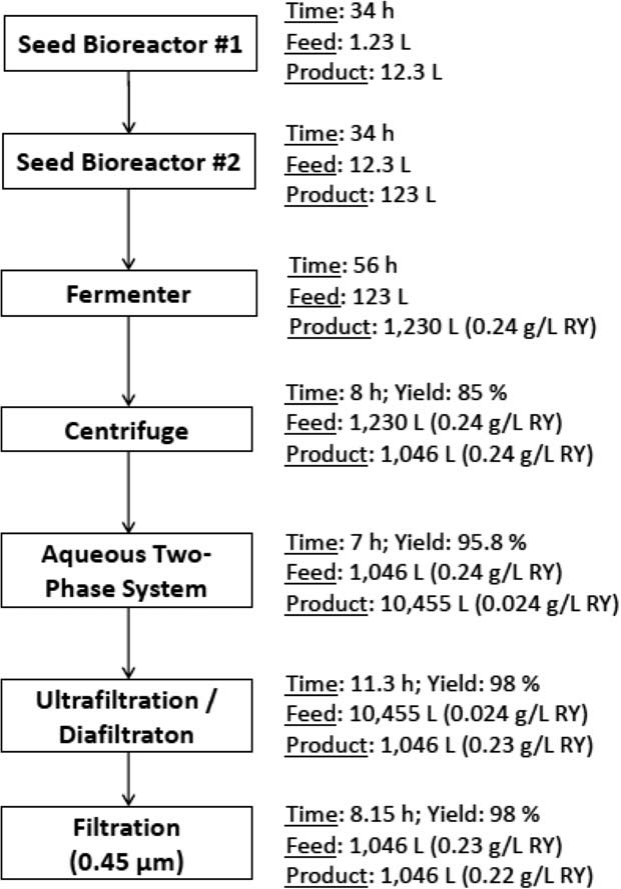
Sequence of unit operations for the production of Royalactin. Each unit operation contains process time, volume‐in and out, yield and concentration of Royalactin.

This section will explain the construction of the base scenario used to design the bioprocess for the production and recovery of Royalactin. First, the sequence of unit operations was proposed following literature,^17^ from it the titer (0.242 ± 0.134 g/L) and ATPS recovery yield (95.8 ± 1.1%) were also obtained. It was decided to design the bioprocess only analyzing ATPS, as it is the only unit operation for which there is evidence that selective recovery of Royalactin is possible. The bioprocess is presented as a flowchart in Figure 1.

To determine the size of the bioprocess unit operations a desired target output has to be proposed. Data have shown that human consumption of Royalactin can decrease cholesterol,^11^ but also can increase the rate of cell division and prevent cell death.^9^ A potential vehicle for the distribution of Royalactin is in a sports beverage, especially in a recovery drink, for the prevention of muscle loss and increased repair. We used this as the bioprocess target output. According to the literature,^25^ one top selling brand of recovery drinks accounts for 4 billion litres per year globally (nearly 50% of the market share in 2011), with the main competitor brand selling a little over 2%.^26^ Taking into account the entry barriers to existing markets, it was decided to design the bioprocess conservatively to capture a 0.5% of the market share of this main competitor (0.0128% of the total market share). The final decision to estimate the annual production is the concentration in each product, it was fixed at 50 mg/L. This value was obtained from analyzing Royalactin doses to different organisms,^2^, ^9^ including human,^3^, ^4^ and taking the half of the lower concentration used.

Labor level was an important parameter to establish. Labor should be between 10 and 15% of the production cost,^19^ the number of employees was assigned to be on 13%. The operator wage was set according to the current wages in the United Kingdom.^27^ Quality control (QC) costs were modified from the default values assumed by BioSolve, since this software assumes QC costs are based upon monoclonal antibody (Mab) production which is a far more exacting process of manufacture. A feature of BioSolve is the large library of equipment and materials which facilitates rapid economic analysis. Costs of the material that are not available can be input by the user. For this paper, the only inputs were recipes for media and buffer, particularly for those during fermentation and recovery by ATPS.

After setting up the model, an initial estimate of CoG/g was obtained as the "Base Case Scenario". The CoG/g for this was of US$ 843.

**Figure 2:**
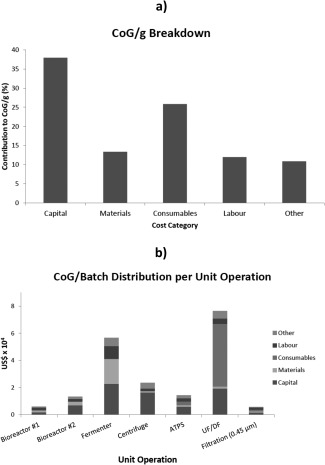
Deterministic analysis results. (a) Cost of goods breakdown by cost categories. (b) Cost of goods per batch distributed per unit operation.

BioSolve also has the capacity of showing the breakdown of the CoG/g. For the Base Case Scenario this is shown in Figure 2. Here it can be seen that the main contribution to the costs comes from the capital, followed by the consumables (Figure 2a). The consumables costs are in turn dominated by the ultrafiltration/diafiltration (UF/DF) step (Figure 2b). This is because of the large quantities of materials needed to perform the ATPS operation which the UF/DF step must subsequently process.

## Identification of Uncertainties/Sensitivity Analysis

In the real world, parameters like titer, process recovery yield, target output, materials cost and operator wages can all change. All affect the CoG/g to some extent, but some have a bigger influence than others. It is important to determine which are the key parameters in order to develop a strategy to control them in order to reduce CoG/g. To identify these parameters a sensitivity analysis was performed, in which a group of process parameters were increased and decreased systematically to analyze the effects on the CoG/g. The parameters used were selected based on the literature.^20^, ^21^, ^28^ Table 1 presents these parameters, along with the corresponding values for a "Worst Case Scenario" and a "Best Case Scenario".

**Table 1:** Scenarios Used for Sensitivity Analysis

	Scenarios		
Variable	Worst	Base	Best
Fermentation titer (g/L)	0.108	0.242	0.376
DSP yield (%)	94.7	95.8	96.9
Material cost (%)	+25	0	−25
Target output (kg/year)	12.8	25.6	51.2
Operator wage (location)	US	UK	Mexico
*Production operator*	*$41,872*	*$32,935*	*$37,689*
*Production supervisor*	*$52,337*	*$38,490*	*$37,689*
*Quality assurance*	*$70,149*	*$56,176*	*$35,995*
*Quality control*	*$39,101*	*$31,400*	*$35,995*

For the fermentation titer and DSP yield the range of values was set by the mean plus and minus one standard deviation.^14^ Materials costs are typically reported to change in a range of ±25%,^20^, ^21^ and this was adopted in the current study. The 25% change in material costs was applied to a subset of the dominant consumables which, for this process, are the fermentation media (buffered glycerol–complex medium) and the UF/DF filters.

**Figure 3:**
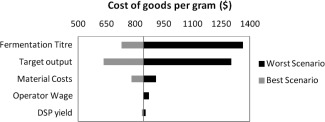
Cost of goods sensitivity to different parameters, vertical axis crosses at the base scenario (CoG/g = $843.00).

Target output variation was determined by making the base case scenario target double or half. Finally, the operator wage was adjusted to the prevailing pay rates in two countries that have a higher and lower salary than United Kingdom. The United States (US) was chosen because it has a large number of biotechnological companies, including a great number of suppliers. Mexico offer lower wages than the other two countries. Each value on Table 1 was input into the BioSolve model individually and the corresponding CoG/g was recorded. Figure 3 show the results for the sensitivity analysis.

From Figure 3, it can be seen that fermentation titer, target output, and the material costs are the top three parameters affecting the CoG/g. It is important to note that when fermentation titer was better than the base case scenario it had less impact on the CoG/g compared to when it was below the base case conditions. These results show that it is important to design a strategy that achieves a CoG/g below corresponding to the base case scenario. To this point the insights have all been derived from individually modifying each parameter, but during a real process, all parameters can change simultaneously. It is also important to combine the impact of uncertainty on these changes when the occur simultaneously in order to assess the overall impact on likely manufacturing scenarios.

## Monte Carlo Analysis

A Monte Carlo analysis was performed in order to understand how the CoG/g changes when the three key parameters (fermentation titer, target output, and material costs) were allowed to vary simultaneously, reflecting real manufacturing behavior. BioSolve is not currently capable of performing this type of analysis and therefore a program in Visual Basic was coded to generate random values according to a triangular distribution. This type of probabilistic distribution is often applied for bioprocess parameters with an expected minimum, maximum, and most likely value.^22^, ^29^ The limits for each function were taken from the values used for the sensitivity analysis. A moving average (MA) was calculated after each simulation run. Stable outcomes were achieved after 300 simulations runs and this was adopted as the standard for the Monte Carlo analysis.

**Figure 4:**
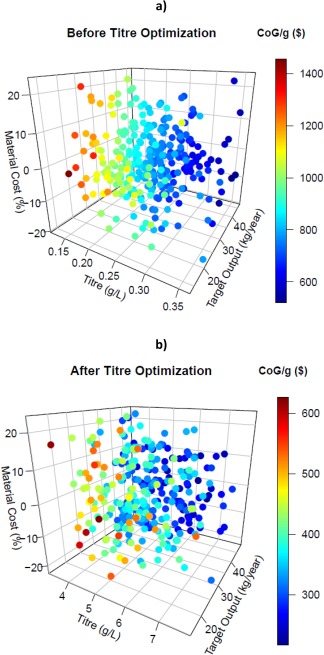
**Monte Carlo Analysis results for cost of goods after generating random values for** titer**, target output, and material cost**. Cost of goods is represented by color variable. (a) CoG/g before titer optimization (titer: 0.242 ± 0.134 g/L; CoG/g: mean = $519.80, median = $821.58, range = $936.47). (b) CoG/g after titer optimization (titer: 5.44 ± 2.44 g/L; CoG/g: mean = $359.75, median = $347.23, range = $409.70).

Of the three parameters to be analyzed, only titer can be optimized experimentally. In order to analyze the effect of optimizing this parameter, a certain base value needed to be proposed. Based on literature,^17^ one strategy to increase the fermentation titer is by strain selection, which can enhance production by threefold.^30^ Alternatively, the incorporation of different types of vector can increase titer. For example plasmids containing zeocin resistance (pPICZ A‐C) have been reported to yield a hyper‐resistant strain of *Pichia pastoris*.^31^ Increasing gene copy number can multiply production up to 7.5‐fold.^32^ If both strategies were to be applied simultaneously, an increase in fermentation titer up to 22.5‐fold might be theoretically expected. This analysis was used to provide a new titer range: 5.44 ± 2.44 g/L. Monte Carlo analysis was then performed for two scenarios, before and after titer optimization. Figure 4 presents the results for the 300 simulation runs for both scenarios.

Figure 4a shows that to achieve a low CoG/g it is necessary to achieve high fermentation titer, high target output, and low material costs. The density of the computed simulations progresses from low titer, low target output and high material costs (conditions that yield high CoG/g) to high titer, high target output, and low material costs (conditions necessary for reduced CoG/g). After optimization of titer (Figure 4b), the simulation results change quite significantly with material costs having little influence and the dot density goes from low target output and low titer (elevated CoG/g) to high target output and high titer (low CoG/g) without noticeable influence of material costs. It is important to note that titer now has less effect than target output.

After obtaining the values for the CoG/g, a linear model was calculated relating CoG/g as a function of titer, target output and material costs (Table 2). It can be seen that after optimization of the fermentation titer, material costs are no longer statistically significant (*α* = 0.01). This is consistent with the dataset in Figure 4. For bioprocesses that achieve a low level of production, the main focus is on the improvement in the process parameters (e.g., fermentation titer). Here is important to ensure that the titer is not low because CoG/g increases rapidly. After optimization, the rank order of the parameters changes (Table 3), and target output now becomes the parameter that influences most the CoG/g. Strikingly then the consequences of optimization of the bioprocess is to shift the manufacturing strategy, which a potential company might pursue, from being process‐oriented to product‐oriented and a strategy that seeks to capture market share needs to be implemented.

**Table 2:** Linear Models for CoG/g in Terms of Fermentation titer, Target Output, and Material Costs

	Before titer Optimization		After titer Optimization	
Parameter	Coefficient	*p* value	Coefficient	*p* value
Intercept	1727.84	<2 × 10^−16^	722.76	<2 × 10^−16^
Fermentation titer	−1881.71	<2 × 10^−16^	−11.42	2 × 10^−15^
Target output	−14.383	<2 × 10^−16^	−10.01	<2 × 10^−16^
Material costs	2.61	3.19 × 10^−14^	0.18	0.17

**Table 3:** Values for each Bioprocess Parameter Analyzed by Monte Carlo Simulation After Optimization of Fermentation Titer

Bioprocess Parameter	Statistic Parameter	Value ($)
Target output	Mean	366.63
	Standard deviation	83.73
Titer	Mean	388.74
	Standard deviation	13.50
Material costs	Mean	382.90
	Standard deviation	1.23

## Conclusions

This paper sets out an economic analysis performed using the commercial software, BioSolve, to analyze potential manufacturing strategies for the production of Royalactin, an interesting protein with valuable properties for potential use as a dietary supplement. Contrasting the order of importance of parameters before and after optimization provided insight on how a company strategy might change in the light of different manufacturing scenarios. The need to conduct economic analysis incorporating the impact of uncertainties by performing Monte Carlo simulation runs is demonstrated, such analysis allows the user to determine how the CoG/g are distributed and for a range of possible scenarios to be investigated which work to reduce the CoG/g. This study provides a framework for future attempts to produce Royalactin at scale. Analysis of process sensitivities was carried out for two scenarios, before and after titer optimization. Results show that work to improve the fermentation titer needs to continue in order to obtain a low‐cost product.
